# Establishment of a droplet digital PCR detection method for Vp4 gene of PoRV

**DOI:** 10.3389/fvets.2026.1742171

**Published:** 2026-02-09

**Authors:** Xinwei Tan, Xiaoyu Zheng, Guangyuan Zou, Manxin Ma, Ze Yuan, Gong Lang, Guihong Zhang

**Affiliations:** 1 Guangdong Provincial Key Laboratory of Zoonosis Prevention and Control, College of Veterinary Medicine, South China Agricultural University, Guangzhou, China; 2 Key Laboratory of Animal Vaccine Development, Ministry of Agriculture and Rural Affairs, Guangzhou, China; 3 Zhaoqing Branch Center of Guangdong Laboratory for Lingnan Modern Agricultural Science and Technology, Zhaoqing, China

**Keywords:** clinical diagnosis, DDPCR, detection method, PoRV, VP4

## Abstract

Diarrhea outbreaks in pigs occur most frequently during winter, porcine rotavirus (PoRV) is one of the important diarrheal diseases. Droplet digital polymerase chain reaction (DDPCR), a detection method that can perform absolute quantification of genes. The study aimed to diagnose PoRV infection using a probe-based DDPCR. 10 2-day-old piglets with mild diarrhea were obtained from a commercial pig farm. No PoRV was detected in the anal swab using colloidal gold test strips. To verify the results of the colloidal gold test strips, RT-qPCR was performed, which identified PoRV in six piglets. Given the limited sensitivity of colloidal gold test strips and RT-qPCR, we developed a DDPCR assay targeting the PoRV Vp4 gene for enhanced detection. The DDPCR assay demonstrated optimal performance at a primer:probe concentration of 400:400 nM and an annealing temperature of 57 °C. It achieved a minimum detection limit of 0.21 copies/μL, the detection sensitivity has been enhanced by 100 times compared to RT-qPCR. Using the established DDPCR detection method, the four samples that tested negative by RT-qPCR were re-tested, and all were found to be PoRV-positive, indicating that the sensitivity of DDPCR was higher than that of RT-qPCR. This study highlights its potential as a valuable tool for early clinical diagnosis and disease control in piglets.

## 1 Introduction

Porcine viral diarrhea is the main cause of high mortality in piglets and poses a serious threat to the global pig industry (1). Porcine rotavirus (PoRV) was first isolated from pigs in the UK in 1974 (2). Its clinical symptoms include watery diarrhea, vomiting, and anorexia. PoRV is an intestinal pathogen that causes diarrhea in newborn piglets, with a mortality rate as high as 95% (3). As pigs grow older, their resistance gradually increases, and adult pigs usually show asymptomatic infections. Pregnant sows transmit the virus to piglets through the placenta. Concerningly, PoRV can spread between pigs and humans, posing a significant threat to public health (4). Research has shown that PoRV ranked as the second most common viral cause of pig diarrhea, with a prevalence rate of 25.81–50.81% in the samples and a farm prevalence rate of 72.77% (5).

PoRV is a non-enveloped, double-stranded RNA virus and a member of the genus Rotavirus of the Reoviridae family. Its genome is approximately 18,500 bp in length and consists of 11 dsRNA segments encoding 6 structural proteins (Vp1–Vp4, Vp6, and Vp7) and 6 non-structural proteins (NSP1–NSP6). PoRV is classified into seven serotypes (A–J) based on Vp6 (6). The structural proteins Vp7 and Vp4 that determine the genotype jointly form the outer shell of RV, and are important antigens for neutralization and the induction of neutralizing antibodies (7). Vp4 is the outer shell protein of PoRV and is responsible for the part of receptor attachment during infection. The Vp4 protein is also a potential vaccine immunogen against PoRV infection, as it can induce the animal body to produce specific neutralizing antibodies and generate systemic immunity in piglets (8). Owing to the complex mixed infection situation, high variability, and global prevalence of PoRV, it has gradually become an important pathogen that causes diarrhea in pigs (9). Therefore, there is an urgent need for rapid and accurate diagnostic techniques for PoRV to enable the immediate implementation of effective measures for prevention and control after diagnosis, thereby reducing economic losses in the pig industry.

Various methods are available for the detection of PoRV, including viral isolation, indirect immunofluorescence, real-time qPCR (RT-qPCR), and enzyme-linked immunosorbent assay. RT-qPCR has been widely used for the identification and typing of PoRVs because of its relatively high sensitivity and specificity for target genes. It has been reported that the minimum detection sensitivity of RT-qPCR for PoRV is 6.0 × 10^1^ copies/μL (10). However, RT-qPCR detection relies on the establishment of standard curves and Cq threshold lines, which may lead to human error. Considering the complexity and large sample volumes, the sensitivity of this method is limited by multiple detection factors. Therefore, establishing a more accurate method for the early detection of PoRV infections is necessary.

The traditional detection methods face two major challenges in the diagnosis of PoRV. Firstly, there are often inhibitors such as polysaccharides and hemoglobin in pig intestinal samples, which can easily lead to false-negative results. Secondly, the viral load is extremely low during the early stage of infection, and conventional methods are difficult to achieve precise detection before symptoms appear. Droplet digital polymerase chain reaction (DDPCR) disperses the samples into tens of thousands of independent reaction units through microdroplet partitioning technology, effectively diluting the inhibitors in the samples and significantly enhancing the anti-interference ability (11). DDPCR is a detection method that perform absolute quantification of genes without the need for a standard curve. During the detection process, a sample be divided into up to 20,000 individual water-in-oil droplets, and each sample undergo thousands of PCR amplifications (12), with the viral content in each droplet evaluated separately (13). Therefore, the impact of potential differences in amplification efficiency and amplification inhibition in the samples on the viral quantification results is relatively small. The sample distribution process also effectively concentrates the template in micro-reactions, improving the sensitivity of trace template detection by reducing competition for amplification reagents among different targets in the reaction mixture (14). The Poisson algorithm is used to calculate the concentration of positive droplets in the sample and perform absolute quantification, eliminating the need for a standard curve (15). Compared to RT-qPCR, DDPCR offers advantages such as absolute quantification, less influence from the detection system, and high sensitivity (16). DDPCR has been widely applied in many fields such as food certification, identification of genetically modified organisms, and clinical testing (17–19). It is reported that currently in China, a DDPCR detection method for PoRVA has been developed based on the conserved sequence of the VP6 gene, and its minimum detection limit is 3.97 copies/μL (20).

Therefore, in this study, a probe-based DDPCR was developed for the Vp4 gene of PoRV to diagnose PoRV infection. This detection method, which exhibits high sensitivity and specificity, provides an effective approach and scientific basis for early differential diagnosis of PoRV in the pig industry.

## 2 Materials and methods

### 2.1 Ethical approval

All animal experiments were approved by the Animal Ethics Committee of the South China Agricultural University (Ethics No. 2025C024) and followed the Guiding Principles for Animal Biomedical Research.

### 2.2 Using colloidal gold test strips to detect samples

The swab was insert into the piglet’s anus 1–1.5 cm, rotate it 4 times, stay for 15 s, immerse it in the extraction solution, rotate and squeeze it 5 times, stay for 30 s, and discard the swab. The sample solution was processed by a dropper to draw and vertically add 3–4 drops to the sample addition hole of the test strip. It was placed flat on the table and wait for 10–20 min as per the instructions.

### 2.3 Preparation of the recombinant PoRV Vp4 standard plasmid and viral genome

Primers and probes were designed based on the Vp4 sequence of PoRV (GenBank: OQ504196.1) in NCBI to amplify the Vp4 gene, and were cloned into the pUC57 vector, named pUC57-PoRV-Vp4. The primers used are listed in Table 1. Genomic RNA was extracted from the samples using an AxyPrep Body Fluid Virus RNA Miniprep Kit (Axygen, USA), and the extracted genomic RNA was reverse transcribed into cDNA using a SartScript III RT Kit (Genstar, China). The genomes of PRRSV, SIV, ASFV, PEDV, PDCoV and PCV2 used in this study were stored at the Department of Infectious Diseases, South China Agricultural University.

**Table 1:** The sequence of primers and probes.

Primer	Sequence (5′ → 3′)	Product length
PoRV-Vp4-F	GAGGTCACTGGCAGCTGATT	111 bp
PoRV-Vp4-R	TGTAACGATACAGCGCCTCC	
PoRV-Vp4-Probe	FAM-AGTAATCTGTAATGGTGGTAGCTATA-MGB	

### 2.4 Design of primers and probes

In this study, based on the highly conserved region of the PoRV Vp4 gene sequence in GenBank, specific primers and probes targeting the PoRV Vp4 gene were designed using the Oligo 7 software (Table 1) and synthesized by Sangon Biotech (Shanghai) Co., Ltd. for RT-qPCR and DDPCR detection. The process of designing primers and probes are as follows: Start the software and import the sequence, select the design type and set the parameters, locate and search for primers, evaluate and optimize the primers, design probes, export and save.

### 2.5 Preparation of the standard curve

The standard plasmid pUC57-PoRV-Vp4 was serially diluted 10-fold over eight gradients (10^−1^–10^−8^) to generate a standard curve for RT-qPCR and to determine the standard curve for DDPCR (QX200 Droplet Digital PCR System, Bio-Rad). The reaction system used for DDPCR is shown in Table 2.

**Table 2:** The reaction system of DDPCR.

Component	Volume (μL)
ddPCR™ Supermix for Probes (No dUTP)	10.0
PoRV-F	0.4
PoRV-R	0.4
PoRV-Probe	0.4
Template cDNA	2
ddH_2_O	6.8

Droplets were generated using the QX200 droplet generator (Bio-Rad), and 20 μL of droplets were transferred to a 96-well plate and sealed for PCR under the following conditions: 10 min at 95 °C, followed by 45 cycles of 30 s at 94 °C, 1 min at 57 °C, and 10 min at 98 °C. All steps were performed at a heating rate of 2 °C/s. Droplet fluorescence was analyzed using the QX200 droplet reader with QuantaSoft software (QuantaSoft software v1.7, Bio-Rad). Template-free samples were used as negative controls to monitor false-positive contamination and primer dimer formation.

The same primers and probes used for DDPCR were used for RT-qPCR. The copy number of the pUC57-PoRV-Vp4standard plasmid was calculated using the following formula: copy number (copies/mL) = (6.02 × 10^23^) × (g/mL) / (DNA length × 660). The copy numbers of the RT-qPCR samples were calculated based on a standard curve. The qRT-PCR system is listed in Table 3.

**Table 3:** The reaction system of RT-qPCR.

Component	Volume (μL)
2 × AceQ Universal U + Probe Master Mix V2	10.0
PoPV-F	0.4
PoRV-R	0.4
PoRV-Probe	0.2
Template cDNA	2
ddH_2_O	7

The reaction conditions for RT-qPCR are as follows: 37 °C for 2 min, 95 °C for 5 min, followed by 45 cycles of 95 °C for 10 s and 57 °C for 30 s.

### 2.6 Sensitivity and specificity evaluations of RT-qPCR and DDPCR

To evaluate the sensitivity of the primers and probes for the PoRV Vp4 gene, the pUC57-PoRV-Vp4 plasmid was diluted in a gradient and detected via RT-qPCR and DDPCR. After the standard plasmid was diluted 10 times continuously and diluted in six gradients (10^−7^–10^−12^), sensitivity was evaluated via RT-qPCR and DDPCR. To assess the specificity of the primers and probes for the PoRV Vp4 gene, the genome of seven common six viruses (PRRSV, SIV, ASFV, PEDV, PDCoV, and PCV2) was used as a template, and RT-qPCR and DDPCR were performed using PoRV-specific primers and probes.

### 2.7 Clinical sample testing

To assess the sensitivity and accuracy of DDPCR in detecting low PoRV viral loads, results from RT-qPCR and DDPCR were compared across the four previously collected clinical samples suspected of PoRV infection.

## 3 Results

### 3.1 Colloidal gold test strips and RT-qPCR detection of rectal swabs from piglets

**Figure 1:**
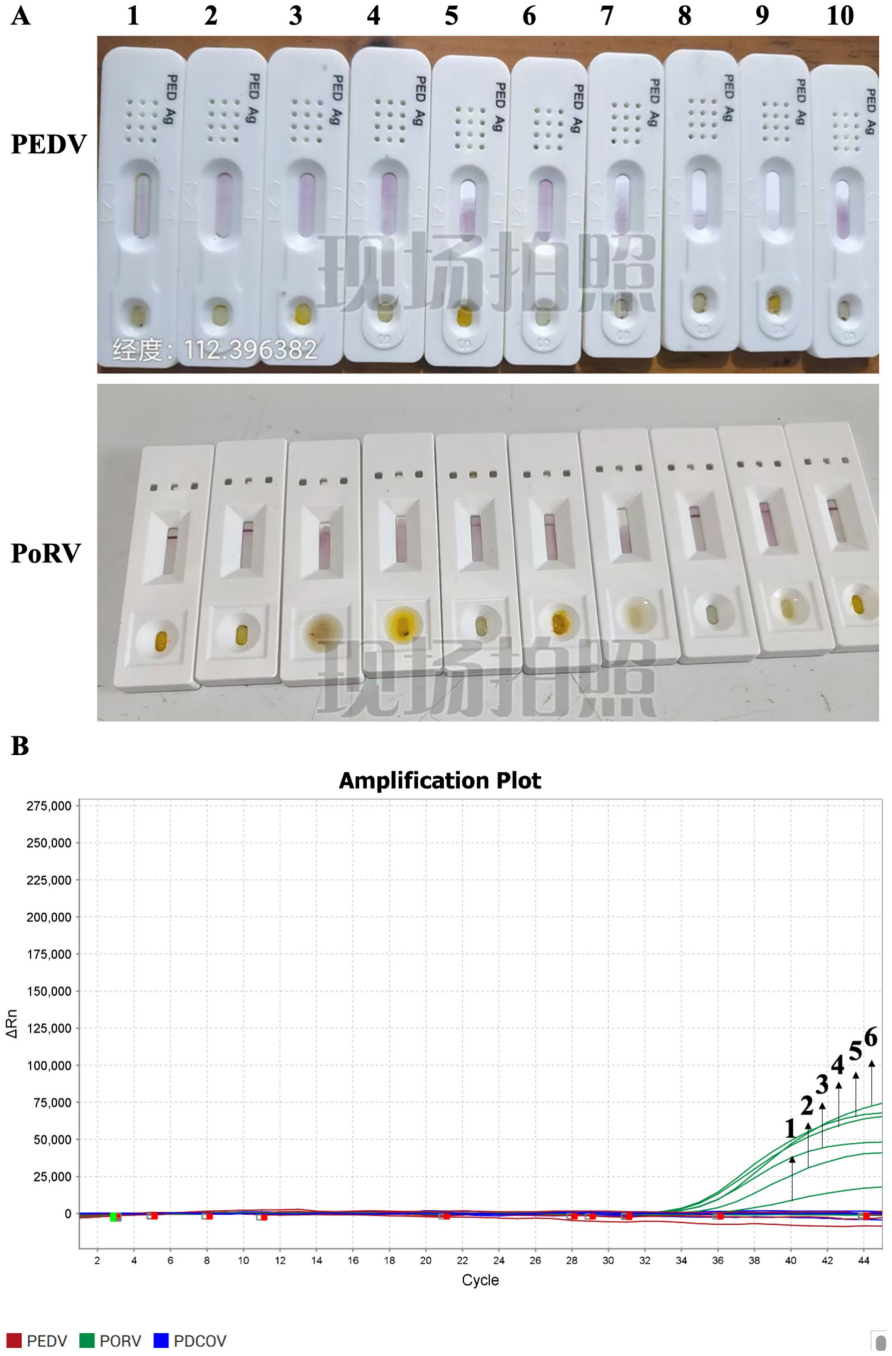
Disease detection was conducted for the infection of PEDV, PoRV, and PDCoV in piglets. **(A)** Colloidal gold test strips were used to detect the antigens of PEDV and PoRV in the anal swabs of piglets. Samples 1–10 represent ten rectal swab tests from ten piglets. All are negative. **(B)** The nucleic acids of PEDV and PoRV in the anal swabs of piglets were detected by RT-qPCR. Samples numbered 1 to 6 are positive for PoRV. Each data represents the results of three independent experiments (mean ± standard deviation).

Ten 2-day-old piglets were purchased from a pig farm in the Guangdong Province. They exhibited mild diarrhea at the beginning of rearing, and the severity of the diarrhea gradually increased. Colloidal gold test strips were used to detect antigen the rectal swabs collected from 10 piglets on day 1 of rearing. Only the control band was observed, indicating that both PEDV and PoRV were absent (Figure 1A). RT-qPCR was used to test the 10 rectal swabs, and 6 of them were positive for PoRV, with CT values of 36.46, 33.605, 34.756, 33.02, 33.267, and 33.42. The remaining samples were negative for both PEDV and PDCoV (Figure 1B). These results indicate that PoRV infection was present in piglets.

### 3.2 Optimization of annealing temperature and primer:probe concentration ratio in DDPCR

**Figure 2:**
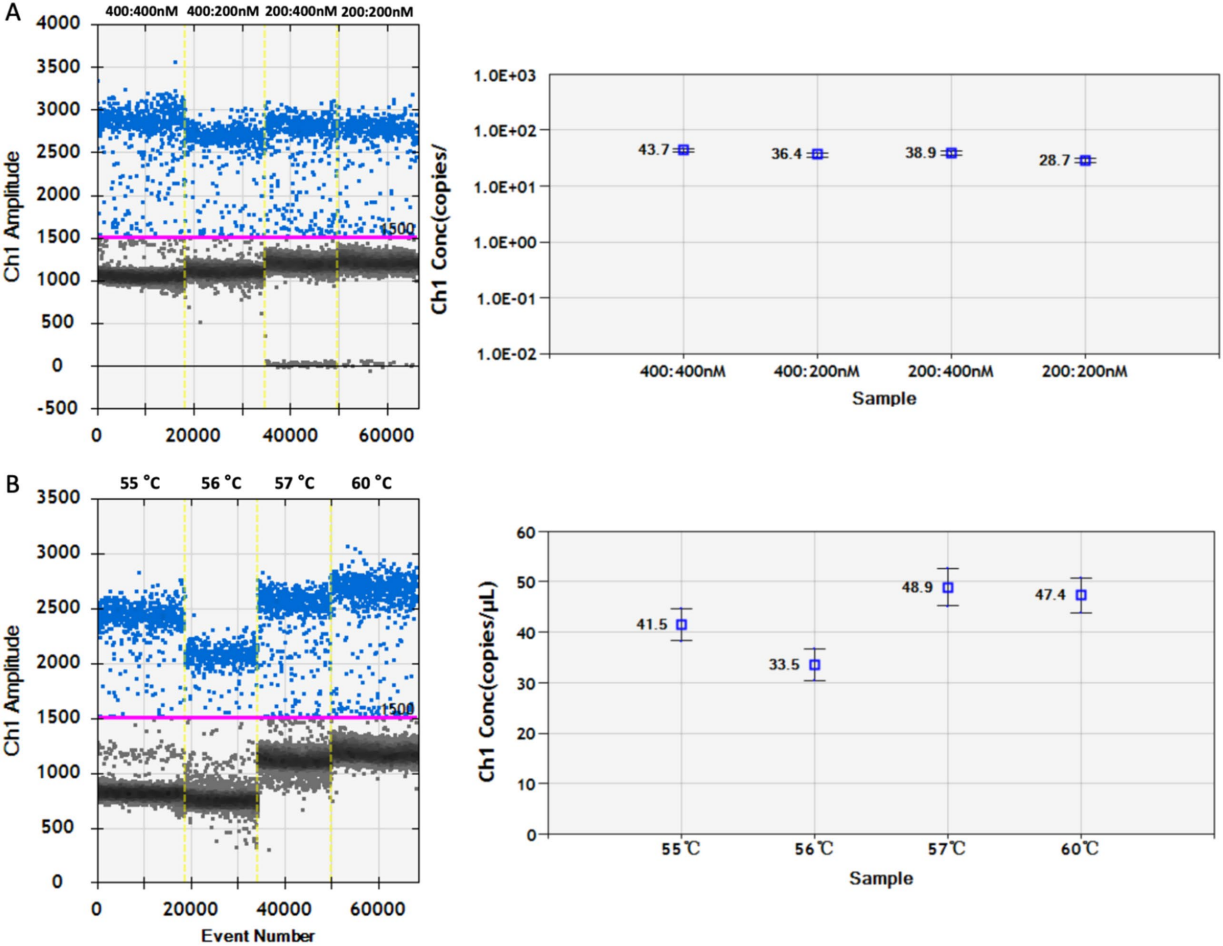
Optimization of annealing temperature and primer-probe concentration ratio in DDPCR program. **(A)** Primers and probes with concentration ratios of 400:400, 400:200, 200:400, and 200:200 was respectively selected for DDPCR detection. The red lines are baselines. **(B)** DDPCR was conducted respectively at annealing temperatures of 55 °C, 56 °C, 57 °C, and 60 °C. All the results were analyzed using the Quanta Soft software. Each data represents the results of three independent experiments (mean ± standard deviation).

The annealing temperature and concentration ratio of the primers and probes were optimized to optimize the DDPCR system for PoRV detection. The primer:probe concentration ratio of the DDPCR detection system was optimized by selecting different concentration ratios for detection. It was found that at a concentration ratio of 400:400 nM, 43.7 copies/μL were detected; at a concentration ratio of 400:200 nM, 36.4 copies/μL were detected; at a concentration ratio of 200:400 nM, 38.9 copies/μL were detected; and at a concentration ratio of 200:200 nM, 28.7 copies/μL were detected. When the ratio of primer:probe concentration is 400:400 nM, the maximum amount of nucleic acid is detected (Figure 2A). Therefore, this concentration ratio is the optimal choice. To select the optimal annealing temperature, DDPCR was performed on PoRV-Vp4 plasmid at annealing temperatures of 55 °C, 56 °C, 57 °C, and 60 °C. The results showed that at 55 °C, 41.5 copies/μL were detected; at 56 °C, 33.5 copies/μL were detected; at 57 °C, 48.9 copies/μL were detected; and at 60 °C, 47.4 copies/μL were detected. At 57 °C, the difference in fluorescence amplitude between the positive and negative droplets was the largest and the maximum amount of nucleic acid is detected, indicating that 57 °C was the optimal annealing temperature (Figure 2B).

### 3.3 Establishment of the standard curve and determination of the sensitivity of DDPCR

**Figure 3:**
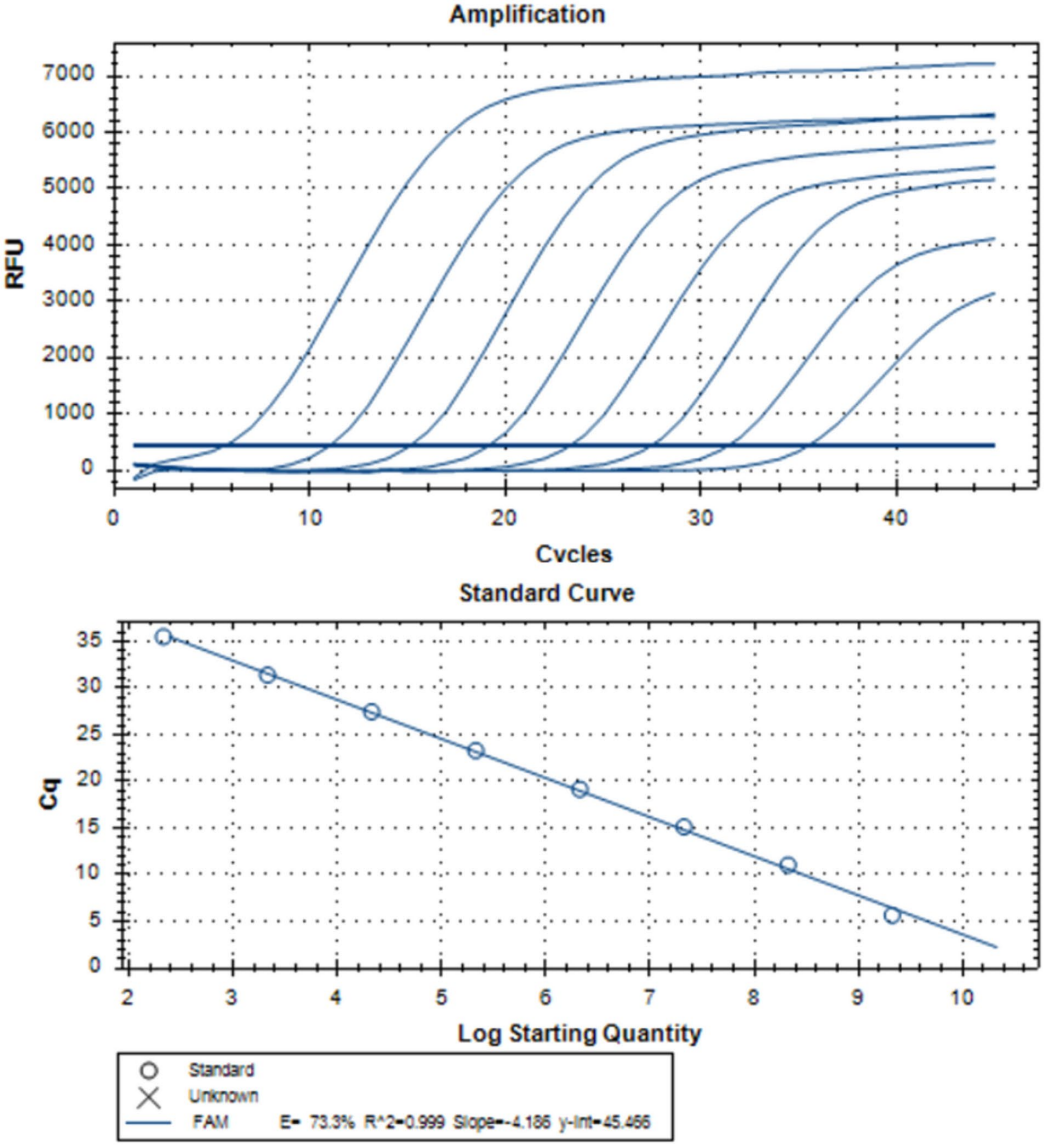
Establishment of the standard curves of RT-qPCR. The positive standard plasmid of PoRV-Vp4 was diluted by a 10-fold concentration gradient (ranging from 2.12 × 10^9^ to 2.12 × 10^2^ copies/μL) and detected by RT-qPCR detection. Each data represents the results of three independent experiments (mean ± standard deviation).

To compare the sensitivity of DDPCR and RT-qPCR assays, a standard curve of RT-qPCR was established using 10-fold serial dilutions of the pUC57-Vp4 plasmid (ranging from 2.12 × 10^9^ to 2.12 × 10^2^ copies/μL), indicating successful construction of the standard curve (R^2^ = 0.999) (Figure 3; Table 4).

**Table 4:** Establishment of the standard curve for RT-qPCR detection.

Standard	CT Value	Copies/μL
Std-1	5.66	2.120E+09
Std-2	11.04	2.120E+08
Std-3	15.13	2.120E+07
Std-4	19.15	2.120E+06
Std-5	23.30	2.120E+05
Std-6	27.46	2.120E+04
Std-7	31.38	2.120E+03
Std-8	35.48	2.120E+02

**Figure 4:**
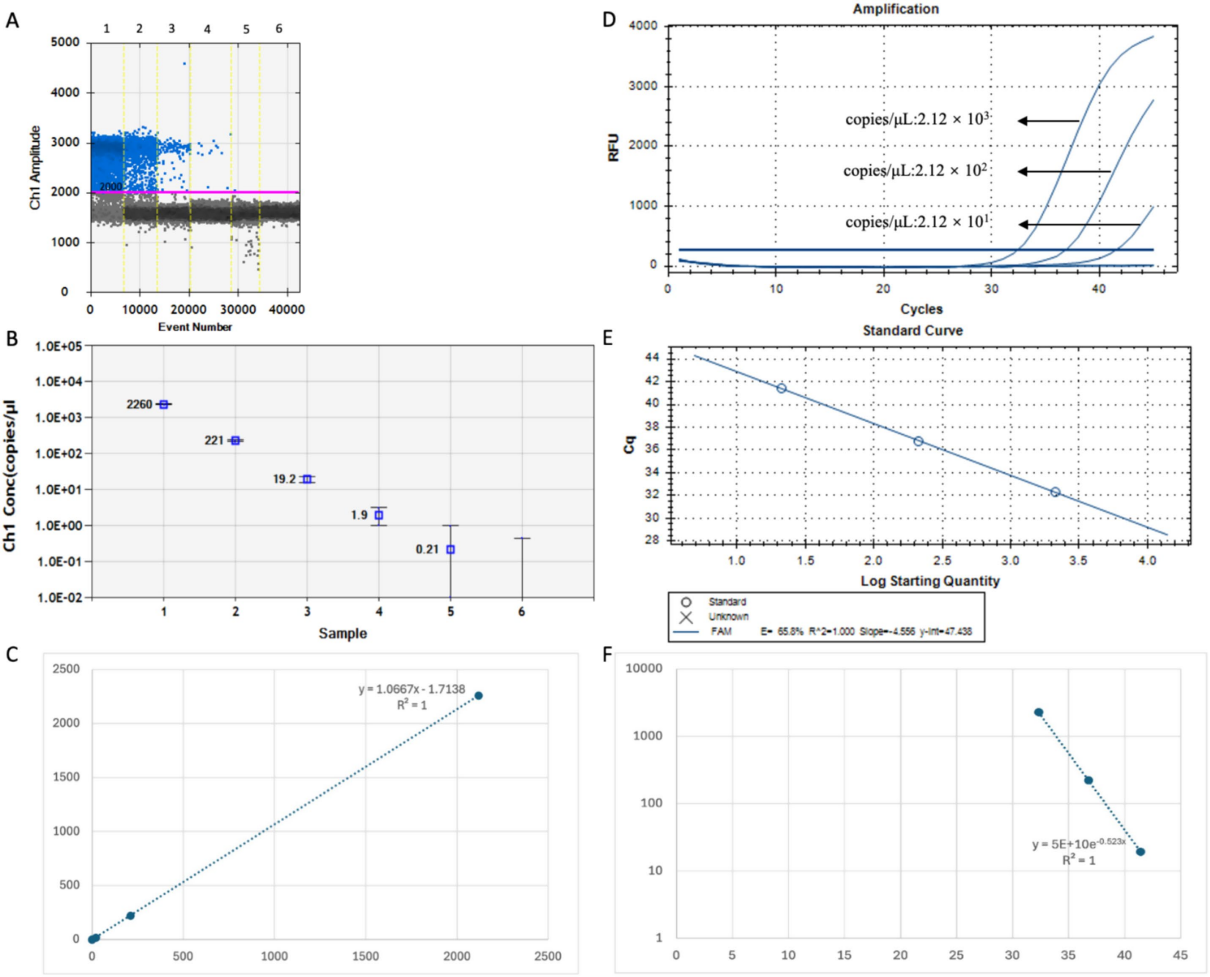
The sensitivity analysis of the standard curves of DDPCR and RT-qPCR. **(A–C)** The PoRV-Vp4 positive standard plasmid was subjected to a 10-fold concentration gradient dilution (10^-7^–10^-12^) and detected by DDPCR. **(D,E)** The PoRV-Vp4 positive standard plasmid was subjected to a 10-fold concentration gradient dilution (10^-7^–10^-11^) and detected by RT-qPCR. **(F)** The linear relationship between DDPCR and RT-qPCR. Both the red line and the blue line are baselines. Each data represents the results of three independent experiments (mean ± standard deviation).

**Figure 5:**
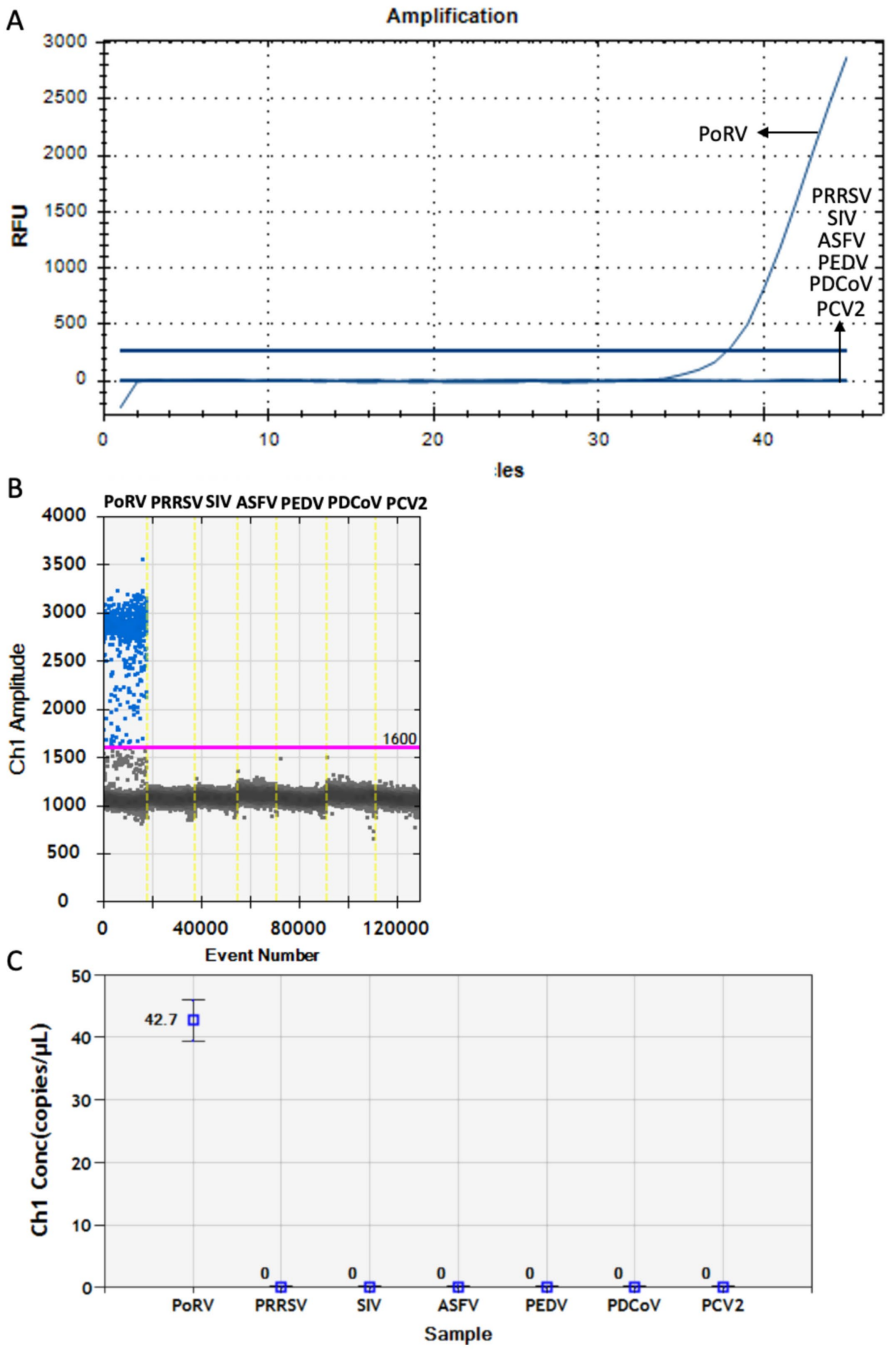
Specific detection of PoRV pathogen by RT-qPCR and DDPCR. **(A)** The nucleic acid of PoRV, PRRSV, SIV, ASFV, PEDV, PDCoV, and PCV2 were detected by RT-qPCR using the primers and probes of PoRV-Vp4. **(B,C)** The nucleic acid of PoRV, PRRSV, SIV, ASFV, PEDV, PDCoV, and PCV2 were detected by DDPCR using the primers and probes of PoRV-Vp4. Both the blue line and the red line are baselines. Each data represents the results of three independent experiments (mean ± standard deviation).

Parallel detection of the standard plasmid pUC57-Vp4 (dilution range of 10^−7^–10^−12^) was performed using RT-qPCR and DDPCR. The results showed that the detection limit of DDPCR was 0.21 copies/μL (Figures 4A,B), whereas that of RT-qPCR was 2.12 × 10^1^ copies/μL (Figure 5B), indicating that DDPCR exhibited higher sensitivity. In this study, both DDPCR (R^2^ = 1) (Figure 4C) and RT-qPCR (R^2^ = 1) (Figures 4D,E) exhibited good linearity according to the regression analysis. The linear relationship between RT-qPCR and DDPCR is good, with an R^2^ value of 1 (Figure 4F).

### 3.4 Specificity detection of DDPCR

For specificity analysis of RT-qPCR and DDPCR, the nucleic acids of PoRV, PRRSV, SIV, ASFV, PEDV, PDCoV and PCV2 were used as reaction template. The results showed that the primers and probes for PoRV Vp4 specifically recognized the PoRV genome and did not produce false-positive results for the other tested samples (Figures 5A–C), thereby confirming the specificity of RT-qPCR and DDPCR detection.

### 3.5 DDPCR detection of clinical samples

**Figure 6:**
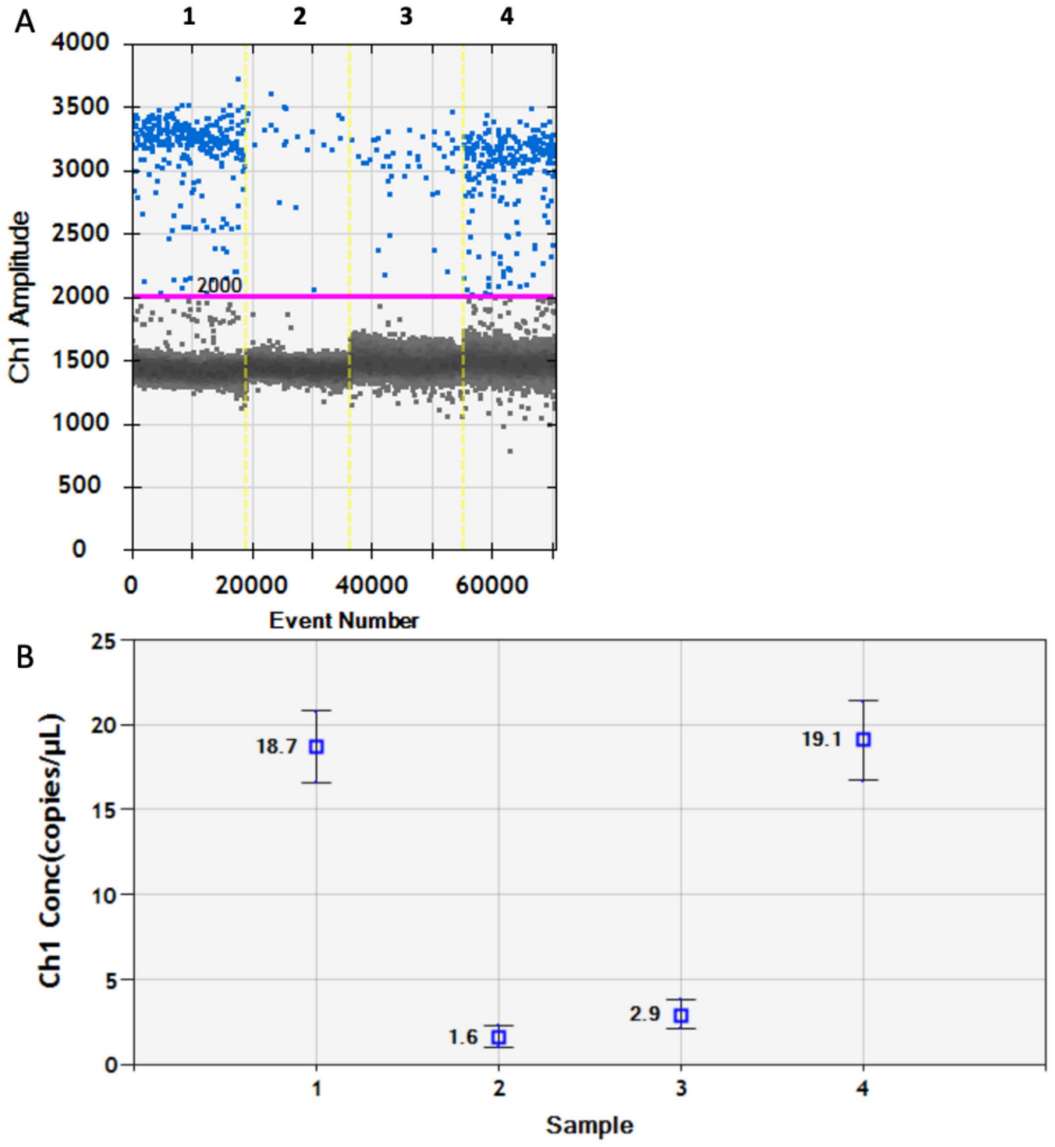
The pathogen of PoRV in the anal swabs of piglets was detected by DDPCR. **(A)** The test results are presented in the form of a dot chart. The red line is baseline. **(B)** The test results presented in terms of copy number. Each data represents the results of three independent experiments (mean ± standard deviation).

DDPCR was then used to analyze four anal swab samples that tested negative by RT-qPCR. PoRV was detected in all four samples, with viral loads of 18.7, 1.6, 2.9, and 19.1 copies/μL, respectively (Figures 6A,B), confirming PoRV infection in each case. Compared with RT-qPCR, DDPCR provides a more sensitive method for the precise quantification of PoRV, especially for the detection of lower concentrations of PoRV. In addition, the DDPCR system quantifies DNA in a highly reproducible manner, without the need for a standard curve. However, we found that the completion time of DDPCR was three times that of RT-qPCR, and the operation of DDPCR was more complex than that of RT-qPCR. The total cost (consumables and labor) of DDPCR was twice that of RT-qPCR. Overall, the DDPCR system was verified as a sensitive and accurate method for the detection of PoRV in clinical molecular virology.

**Figure 7:**
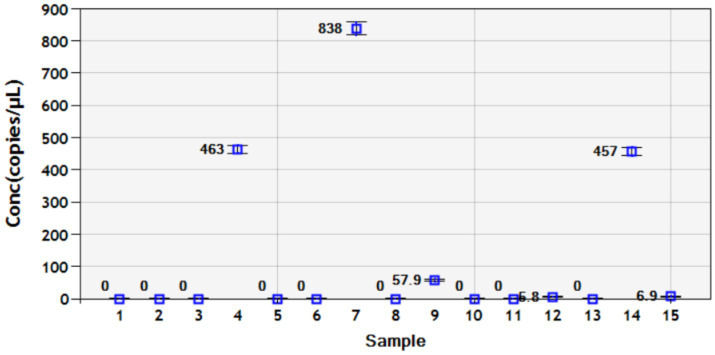
Using the established DDPCR detection method, clinical samples from 15 piglets’ anal swabs were tested. Each data represents the results of three independent experiments (mean ± standard deviation).

To more accurately verify the accuracy and sensitivity of the established DDPCR detection method, 15 clinical samples of anal swabs from piglets collected from different pig farms were tested. The results showed that through DDPCR detection, there were 6 positive samples of PoRV, with virus loads of 463 copies/μL, 838 copies/μL, 57.9 copies/μL, 5.8 copies/μL, 457 copies/μL and 6.9 copies/μL. The reason for the relatively high proportion of PoRV positive infections might be that it is currently winter, which is the high-incidence season for piglet rotavirus (Figure 7). Therefore, it suggests that each pig farm should strengthen the prevention and control measures for diarrhea diseases in piglets during winter.

## 4 Discussion

Animals serve as potential reservoirs for the genetic and antigenic diversity of human rotaviruses, significantly impacting public health. Interspecies transmission and genetic rearrangement events involving human and animal rotaviruses are common in domestic animals, including cattle, cats, dogs, and pigs, and are helpful for studying the genetic diversity and evolution of rotaviruses (4, 21). PoRV infection is the second-leading cause of diarrhea in piglets, second only to PEDV infections (22). Currently, there are no effective measures available for treating PoRV infection; therefore, there is an urgent need to develop a rapid, sensitive, economical, and high-performance method for the early detection of PoRV infection. Vp4 is the outer capsid protein of PoRV, responsible for the part that attaches to the receptors during the infection process. The Vp4 protein is also a potential vaccine immunogen against PoRV infection, as it can induce the animal body to produce specific neutralizing antibodies, resulting in systemic immunity in piglets (23). Therefore, Vp4 is of great significance as a target for detection methods.

DDPCR has many advantages over traditional RT-qPCR methods. In RT-qPCR, DNA-binding dyes are used to calculate the amplification products at the end of each amplification, and the intensity of the fluorescent dye attached to the dsDNA is proportional to the PCR amplification products (24). Specific absolute quantification of viral nucleic acids using RT-qPCR requires the establishment of a standard curve obtained from standard plasmids of known concentrations. However, the accuracy of this curve can be affected by operator handling, introducing errors (25). In contrast, DDPCR enables absolute quantification without a standard curve, eliminating this limitation (26). Although DDPCR employs the same primers and probes as RT-qPCR, it offers superior sensitivity and repeatability. This advantage stems from two distinct features of the DDPCR droplet method: compartmentalization and data collection from endpoint reactions (27). The commonly used detection methods currently include colloidal gold test strips and RT-qPCR. Colloidal gold test strips are suitable for rapid screening and on-site testing. Their advantages lie in their high speed, low cost, and simple operation, but their sensitivity and specificity are relatively low (28). RT-qPCR is suitable for clinical diagnosis, routine laboratory testing, and epidemiological investigations. It strikes a balance between sensitivity, specificity, and cost, and is currently the mainstream nucleic acid detection method (29). DDPCR is suitable for detecting low viral load samples, precise quantification, and complex sample analysis. It has advantages in early infection diagnosis, but it has high costs and long processing time (30). Based on the current findings, the high sensitivity of this method outweighs its limitations of relatively high cost and prolonged processing time in applications such as swine herd purification, introduction quarantine, and early-phase epidemic surveillance. A simple comparison of the reagent and equipment costs for qPCR and DDPCR in each sample has been made, as shown in Table 5.

**Table 5:** The cost comparison of RT-qPCR and DDPCR.

Detection method	Detection limit of PoRV	The estimated reagent costs per sample	The equipment costs	Testing duration
RT-qPCR	1.24 × 10^1^–6.0 × 10^1^ copies/μL	0.3–0.6 $	35,000$	2 h
ddPCR	0.21 × 10^0^ copies/uL	4.5–6 $	215,000 $	3–4 h

Viral infection poses a risk of transmission during the window period, which is the interval between the onset of infection and the detection of the pathogen. Viral shedding often begins before clinical symptoms appear, making early detection reliant on the sensitivity of the diagnostic method. The detection of the virus during the window depends on the type of technology used. Highly sensitive detection can identify viruses faster than traditional methods, thereby shortening the time gap between detection and viral excretion. Compared to that for RT-qPCR, the window period for DDPCR detection is shorter (31). Currently, DDPCR is increasingly used for the diagnosis of viral diseases and is a reliable and amplifiable method for detecting trace amounts of viruses (32). Although DDPCR is still in the early stages of routine clinical implementation, its reliability and sensitivity make it a strong candidate. It has already been applied to detect various DNA and RNA viruses. For example, RT-qPCR can detect cytomegalovirus at a minimum threshold of 3 log10 copies/mL, whereas DDPCR can detect as few as 100 copies/mL (33). The sensitivity of DDPCR for hepatitis virus detection can reach 0.8 copies/10^5^ cells, which is 10–100 times higher than that of RT-qPCR (34). The sensitivity of DDPCR for detecting H1N1 AIV is 30 times higher than that of RT-qPCR, and its accuracy is 10 times higher (35). Furthermore, the sensitivity of DDPCR for SARS-CoV-2 detection is 500 times higher than that of qRT-PCR (36).

Previous studies have reported varying detection limits for PoRV using RT-qPCR-based assays. In the study Establishment and Application of a Triplex Real-Time RT-PCR Assay for Differentiation of PEDV, PoRV, and PDCoV, the minimum detection concentration for PoRV was 6.0 × 10^1^ copies/μL (37). In Establishment and application of a TaqMan-based multiplex real-time PCR for simultaneous detection of three porcine diarrhea viruses, the minimum detection concentration for PoRV was 1 × 10^2^ copies/μL. Similarly, the study Dual priming oligonucleotide (DPO)-based real-time RT-PCR assay for accurate differentiation of four major viruses causing porcine viral diarrhea, the minimum detection concentration for PoRV was 1.74 × 10^2^ copies/μL (38).

In the present study, specific primers and probes were designed for the conserved region of the PoRV Vp4 gene. After optimizing the primer:probe concentration ratio and annealing temperature, a DDPCR method was successfully developed for the detection of PoRV. This study aims to establish a DDPCR detection method for the Vp4 gene of group A rotavirus. Although group A rotavirus is the most common and characteristic rotavirus in pigs, the incidence of group B, C, D and H rotaviruses in pig populations has also been increasing in recent years (39). Therefore, subsequent studies will establish DDPCR detection methods for other groups of rotaviruses, ultimately achieving a universal detection method. The method established in this study has high sensitivity, with a minimum detection concentration of 0.21 copies/μL—100 times higher than that of RT-qPCR. The specificity test results of this method indicated that the primers and probe used did not cross-react with other common porcine viruses (PRRSV, SIV, ASFV, PEDV, PDCoV and PCV2) on pig farms. By comparing the detection sensitivity of DDPCR with that of commercial colloidal gold test strips and RT-qPCR for clinical samples with diarrheal symptoms, the study found that DDPCR could detect positive samples that were not detected by the other two methods. These results indicate that DDPCR offers superior sensitivity.

From the perspective of the Vp4 gene detected in this study, in terms of the potential for cross-species transmission, the Vp4 protein of PoRV has a homology of approximately 65% with that of HRV. The P-type of the Vp4 protein of PoRV A has a higher sequence similarity with HRV. The phenomenon of gene recombination may affect the homology. For example, the Vp4 gene of some PoRV strains may originate from HRV, suggesting the possibility of cross-species transmission (40). Therefore, in the cross-species diagnosis aspect, the DDPCR method established based on the Vp4 gene has the potential to be applicable for detecting different animal rotaviruses. This method still has a long way to go before it can be used clinically.

## 5 Conclusion

This study is the first to establish a DDPCR method for the rapid identification and detection of PoRV Vp4, which not only enhances the accuracy and efficiency of detection but also provides a powerful tool for monitoring, disease prevention and control, and scientific research on PoRV. This detection method will be helpful for conducting epidemiological investigations of Porcine Rotavirus (PoRV) and will make a significant contribution to the prevention and control of viral porcine diarrhea diseases.

## Data Availability

The original contributions presented in the study are included in the article/supplementary material, further inquiries can be directed to the corresponding author.
